# Estradiol is not protective against angiotensin II‐induced hypertension in middle‐aged ovariectomized rats

**DOI:** 10.14814/phy2.70646

**Published:** 2025-11-07

**Authors:** A. P. O. Leite, I. Pires dos Santos, Y. Zha, S. A. Blessinger, H. Petillo, N. Jasti, H. C. Cheeran, R. Menon, A. B. Walker, J. M. Daniel, S. H. Lindsey

**Affiliations:** ^1^ Department of Pharmacology Tulane School of Medicine New Orleans Louisiana USA; ^2^ Tulane Brain Institute New Orleans Louisiana USA; ^3^ Department of Psychology Tulane School of Science & Engineering New Orleans Louisiana USA

**Keywords:** aging, estradiol, hypertension, menopause, renal function

## Abstract

Menopause leads to loss of cardiovascular and renal protection, and while hormone therapy offers benefits, its efficacy may depend on health status at menopause onset. We hypothesized that preexisting hypertension blunts the renal, cardiac, and vascular effects of Estradiol (E2). Female Long‐Evans rats were ovariectomized (OVX) at 46 weeks to model menopause and received either E2 or vehicle, and some were infused with angiotensin II (ANG; 700 ng/kg/min) 4 weeks before OVX. Blood pressure (BP) was measured by tail cuff, renal function by urine collection, collagen deposition by histology, and mRNA expression in aorta and kidney by droplet digital PCR. ANG increased BP and proteinuria (*p* = 0.02), water intake (*p* < 0.001), urinary output, heart weight, and aortic NOX4 (*p* < 0.01), confirming hypertension and oxidative stress. E2 reduced body weight (*p* = 0.02), increased bone mineral content (*p* = 0.01), and prevented uterine atrophy (*p* < 0.001), confirming E2 treatment. While E2 attenuated cardiac hypertrophy (*p* = 0.004), it exacerbated proteinuria, decreased GFR (*p* < 0.05), and failed to reduce aortic NOX4. ANG did not affect tissue estrogen receptor expression, while E2 showed tissue‐specific regulation of GPER and ERα. In this hypertensive OVX model, E2 failed to protect renal and vascular damage, emphasizing the importance of cardiovascular health at menopause when considering hormone therapy.

## Graphical abstract

Estradiol fails to protect in menopausal hypertension.

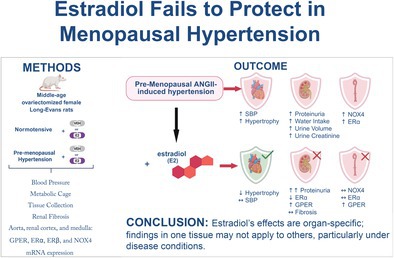

## 1 INTRODUCTION

Hypertension and aging are major risk factors for both cardiovascular and kidney disease, with renal dysfunction often serving as both a cause and a consequence of cardiovascular complications (Zoccali et al., 2023). Chronic kidney disease ranks among the top 10 leading causes of death in the United States and worldwide (Kochanek et al., 2023). Premenopausal women are relatively protected from cardiovascular and renal diseases compared to age‐matched men, an advantage largely attributed to the presence of ovarian hormones, particularly estradiol (Dines & Garovic, 2024). However, this protection diminishes after menopause, highlighting the critical role of sex hormones in maintaining vascular and renal health.

Menopausal hormone therapy (MHT) has been explored for its potential to mitigate postmenopausal cardiovascular risk, but clinical findings remain inconsistent. While some studies report beneficial effects of menopausal hormone therapy on cardiovascular outcomes, others fail to show significant protection (Dines & Garovic, 2024; Manson et al., 2003; Rossouw et al., 2007). These discrepancies have led to the development of the “healthy cell bias” hypothesis, which suggests that MHT is most effective in healthy cells and tissues, whereas its benefits are diminished in the presence of chronic disease such as hypertension (Brinton, 2008). Despite increasing interest, the effects of menopausal hormone therapy—particularly in the context of hypertension and associated renal damage—remain poorly understood in both clinical and preclinical models.

The kidney is one of the most estrogen‐responsive nonreproductive organs, with estradiol influencing glomerular and tubular function, as well as sodium and water homeostasis (Gohar et al., 2020; Singh et al., 2022; Thomas & Harvey, 2023). However, studies investigating estradiol's impact on renal injury have yielded conflicting results. Some suggest estradiol promotes tubular regeneration (Ren et al., 2022), while others report protective effects in healthy kidneys but exacerbation of injury in certain pathological states (Sharifi et al., 2019; Stehman‐Breen et al., 2003). In models of salt‐sensitive hypertension, ovarian hormones are essential for the observed female protection against blood pressure elevation and renal injury (Chappell et al., 2006), yet this protection appears to be lost with aging (Chappell et al., 2008).

While considerable attention has been paid to the protective role of estradiol postmenopause, the influence of pre‐existing hypertension on the impact of MHT remains unclear. In this study, we modeled menopause in the presence or absence of preexisting hypertension to determine whether estradiol retains its protective effects. We hypothesized that hypertension established before menopause would blunt or reverse the beneficial actions of estradiol.

## 2 MATERIALS AND METHODS

### 2.1 Subjects and experimental design

Forty‐eight female Long‐Evans rats (*n* = 48), aged 70 days were obtained from Envigo and allowed to age until approximately 9 months old. The rats were housed in pairs in a temperature‐controlled vivarium, maintained under a 12‐h light/dark cycle, with unrestricted access to food and water unless specified otherwise. After 1 week at the vivarium, all animals were placed on a soy‐free diet ad libitum (Bio‐Serv, Frenchtown, NJ) (20.5% protein, 7.2% fat, 0% fiber, 3.5% ash, and 61.6% carbohydrate).

**FIGURE 1:**
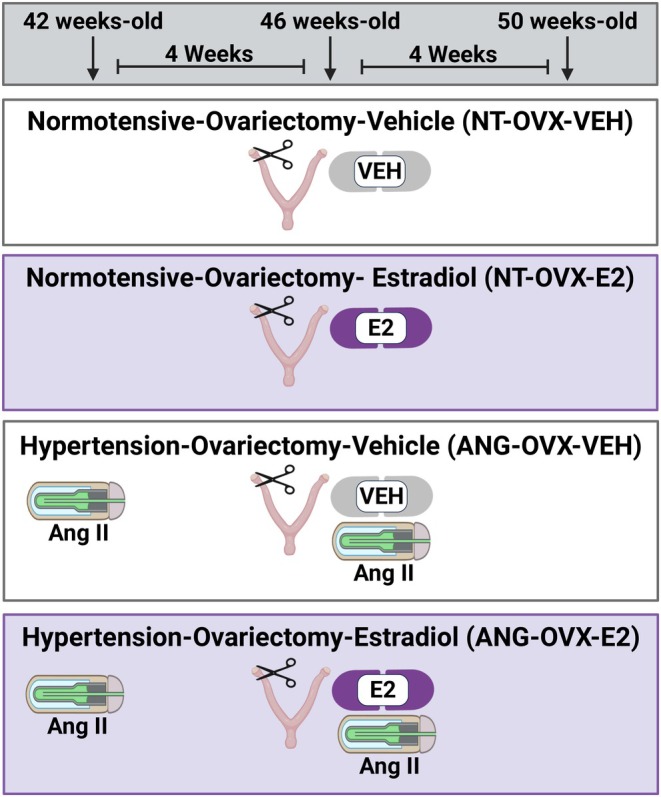
Experimental design. Ovariectomy (OVX) was combined with vehicle (VEH) or estradiol (E2) treatment. Some animals were left normotensive (NT) while some were treated with angiotensin II (ANG), resulting in four groups: NT‐OVX‐VEH, NT‐OVX‐E2, ANG‐OVX‐VEH, and ANG‐OVX‐E2. Created with BioRender.com.

Rats were pair‐housed, with treatments randomized within each cage. Animals were either left normotensive (NT) or infused with angiotensin II (ANG, 700 ng/kg/min, Bachem, #4006473) via osmotic minipump (Alzet 2ML4) 4 weeks prior to ovariectomy (OVX). At 46 weeks, rats underwent OVX and were treated for 4 weeks with vehicle (VEH) or estradiol (E2). At the time of OVX, the pump was replaced with a new four‐week pump containing ANG. This design resulted in four groups: NT‐OVX‐VEH, NT‐OVX‐E2, ANG‐OVX‐VEH, and ANG‐OVX‐E2, *n* = 10–12 per group (Figure 1).

### 2.2 Surgical procedures

Aseptic techniques, postoperative monitoring, and surgical analgesia were implemented. Meloxicam (1–2 mg/kg SC/PO SID) was administered via subcutaneous injection as an analgesic before any surgery. For 2 days after surgery, animals were monitored daily for pain and distress, and additional Meloxicam was given if necessary.

### 2.3 Induction of hypertension

Angiotensin II (ANG, Bachem, 700 ng/kg/min, #4006473) infusion was administered via osmotic minipumps implanted under isoflurane anesthesia. Osmotic minipumps (Model 2004, Alzet, Cupertino, CA, US) were filled with ANG (Sigma) dissolved in saline and incubated at 37°C overnight before implanting intraperitoneally. Pumps were replaced after 4 weeks at the time of OVX, totaling 8 weeks of hypertension.

### 2.4 Ovariectomy and hormone treatment

At 46 weeks of age, considered middle‐aged for this model, animals underwent OVX to reduce circulating endogenous ovarian hormones. They received silastic capsules containing either 25% 17β‐estradiol (Sigma‐Aldrich, St. Louis, MO, #E1024) diluted with cholesterol or 100% cholesterol vehicle. Anesthesia was induced using isoflurane for the procedure, which involved bilateral flank incisions through the skin and muscle wall to remove the ovaries as previously described (Zimmerman et al., 2017). Immediately following OVX, a subcutaneous 5 mm silastic capsule (0.058‐inch inner diameter and 0.077‐inch outer diameter; Dow Corning, Midland, MI, US, #508‐006) was implanted on the dorsal side of the neck. The efficacy of the implants was assessed by measuring uterine weight. Capsules of this size and estradiol concentration sustain circulating estradiol levels at approximately 36 pg/mL, which corresponds to physiological levels typically observed during proestrus (Bohacek & Daniel, 2010).

### 2.5 Tail‐cuff plethysmography

Tail‐cuff plethysmography was used to measure blood pressure in awake rodents using an automated tail‐cuff volume‐pressure recording system (Kent Scientific CODA system). Animals were acclimated to the clear plastic tube restraints for 2 days, and measurements were obtained over three consecutive days to reduce the impact of restraint‐induced stress. Ten to fifteen consecutive measurements were taken for each animal while they were warmed to 35°C, and the systolic blood pressures were averaged over the 3 days.

### 2.6 Metabolic cages

Animals were individually housed in metabolic cages for 48 h, with the first 24 h allocated for adaptation and the following 24 h for water intake and urine collection. Urine samples were centrifuged to remove particulate matter and stored at −80°C until assayed. Urinary protein concentration was determined via Bradford assay (Bio‐Rad, Hercules, CA, #500‐0006) with bovine serum albumin as the standard and is expressed as milligrams of protein per day. Serum and urine creatinine were measured using the Creatinine Assay Kit (QuantiChrom, Swedesboro, NJ, #DICT‐500) following the company's instructions. The creatinine clearance or estimated glomerular filtration rate (eGFR) in milliliters per minute was calculated using the formula (Urine Volume [mL/min] × Urine Creatinine [mg/dL])/Serum Creatinine [mg/dL] as previously described (Zimmerman et al., 2020).

### 2.7 Euthanasia and tissue collection

Rats were euthanized under light isoflurane anesthesia and sacrificed by decapitation. The uterus, kidneys, aorta, and heart were collected and weighed. Tissues for molecular analysis were preserved in RNAlater (Thermo Fisher Scientific, Plaquemine, LA, US, #AM7021) at 4°C for 24 h, then moved to −20°C for long‐term storage. The aorta, kidneys, and heart were formalin‐fixed (10% neutral buffered formalin) overnight and paraffin‐embedded, and 4‐μm sections were mounted onto slides. Samples for histology were kept in 10% neutral buffered formalin (NBF) for 24 h, then stored in 70% ethanol until processed.

### 2.8 Histology

Renal tissue was stained using Masson Trichrome Blue (Newcomer Supply, Middleton, WI, US, #9176A) to analyze collagen deposition and morphology. The aorta was stained with Verhoeff–Van Gieson stain to quantify elastin, aorta area, and diameter (Newcomer Supply, Middleton, WI, US, #9116B). The analysis was performed using Adobe® Photoshop® software. For each tissue section, the entire cross‐section was imaged, and the number of pixels stained with the selected color was counted and expressed as an area fraction (percent of pixels with positive staining within the tissue area). Data analysis was performed by an independent investigator who was blinded to the treatment groups.

### 2.9 Droplet digital polymerase chain reaction

Droplet digital polymerase chain reaction (ddPCR) was accomplished using a previously described method (Hindson et al., 2011; Hutson et al., 2019). Total RNA samples were extracted with Trizol using Qiagen MinElute Columns (QIAGEN, Aarhus, Denmark, #28115). RNA quantity was assessed using a Nanodrop, and samples were stored at −80°C until processing. The ddPCR was conducted using the following validated rat primers obtained from Bio‐Rad: GPER (dRnoCPE5151056), Esr1 (ER‐α, dRnoCPE5176827), Esr2 (ER‐β, dRnoCPE5175914), Sirtuin 1 (Sirt1, dRnoCPE5169220), NOX4 (dRnoCPE5170423), and NOS3 (eNOS, dRnoCPE5171641). The reaction mixture was divided into over 10,000 individual 1‐nL droplets using oil emulsion microfluidics. These droplets were then analyzed with the Bio‐Rad QX200 droplet reader and QuantaSoft software, and the results were converted to copies per nanogram of RNA based on the RNA concentration and the total volume added to the reaction. Samples were either rerun or excluded if they exhibited an excessive number of positive or negative droplets (violating Poisson statistics), had QuantaSoft Quality Scores (Bio‐Rad Laboratories, BioRad, Hercules, CA, US) below 0.85, or contained fewer than 10,000 droplets.

### 2.10 Statistical analysis

Statistical analysis was performed using two‐way ANOVA to evaluate the main effects of ANG and E2 treatment, as well as their interaction. Fisher's Least Significant Difference (LSD) post hoc test was applied to examine the impact of E2 within each condition (normotensive or hypertensive). Because our a priori interest lay with the effect of E2 in the ANG groups, we examined the simple effects of E2. Data that were more than two standard deviations from the mean were excluded from analysis. Data are presented as mean ± SD, and statistical significance was set at *p* < 0.05. Means and standard errors appear in the corresponding figures. Analyses were performed using Prism Version 10.3 software (GraphPad Software, La Jolla, CA).

## 3 RESULTS

### 3.1 Baseline physiological data

Baseline physiological data, including tail‐cuff plethysmography for blood pressure and metabolic cage measurements, were collected before the experiment began, revealing no significant differences between the groups (Table 1).

**TABLE 1:** Baseline physiological data.

	NT‐OVX		ANG‐OVX	
	VEH	E2	VEH	E2
Systolic BP (mmHg)	132 ± 8.3	134 ± 8.7	136 ± 4.2	135 ± 8.9
Diastolic BP (mmHg)	89 ± 9.2	89 ± 8.4	92 ± 6.1	93 ± 2.3
Mean arterial pressure (mmHg)	103 ± 8.5	104 ± 8.3	106 ± 4.9	107 ± 8.5
Pulse pressure (mmHg)	43.4 ± 5.5	44.7 ± 4.1	43.9 ± 5.4	41.5 ± 4.1
Heart rate (bpm)	322 ± 24	311 ± 39	328 ± 33	341 ± 33
Body weight (g)	302 ± 44	298 ± 49	295 ± 26	281 ± 25
Water intake (mL/24 h)	12.5 ± 5.6	14 ± 3.5	14 ± 6	11 ± 6.8
Urine volume (mL/24 h)	3.8 ± 2.4	4.9 ± 3.4	5 ± 4.6	4.2 ± 4.5
Urine creatinine (mg/day)	5 ± 2	6.7 ± 1.9	4.8 ± 1.8	3.8 ± 2.6
Urine osmolality (mmmol/kg)	2034 ± 1400	2083 ± 1264	2563 ± 1411	1596 ± 2022
Proteinuria (mg/day)	2.7 ± 2	3.2 ± 1.7	3.2 ± 2.1	2.2 ± 1.4

*Note*: Data collected prior to any intervention in middle‐aged female rats labeled according to future treatment group: Normotensive (NT) and ANG‐infused hypertensive (HT) rats treated with vehicle (VEH) or estradiol (E2), *n* = 10–12 per group. There were no differences between groups (all *p* > 0.05). Mean ± SD, analyzed by two‐way ANOVA, *n* = 10–12 per group, all *p* > 0.05.

Abbreviations: ANG, angiotensin II; BP, blood pressure; E2, estradiol; NT, normotensive; OVX, ovariectomy; VEH, vehicle.

### 3.2 Blood pressure and tissue weights

**FIGURE 2:**
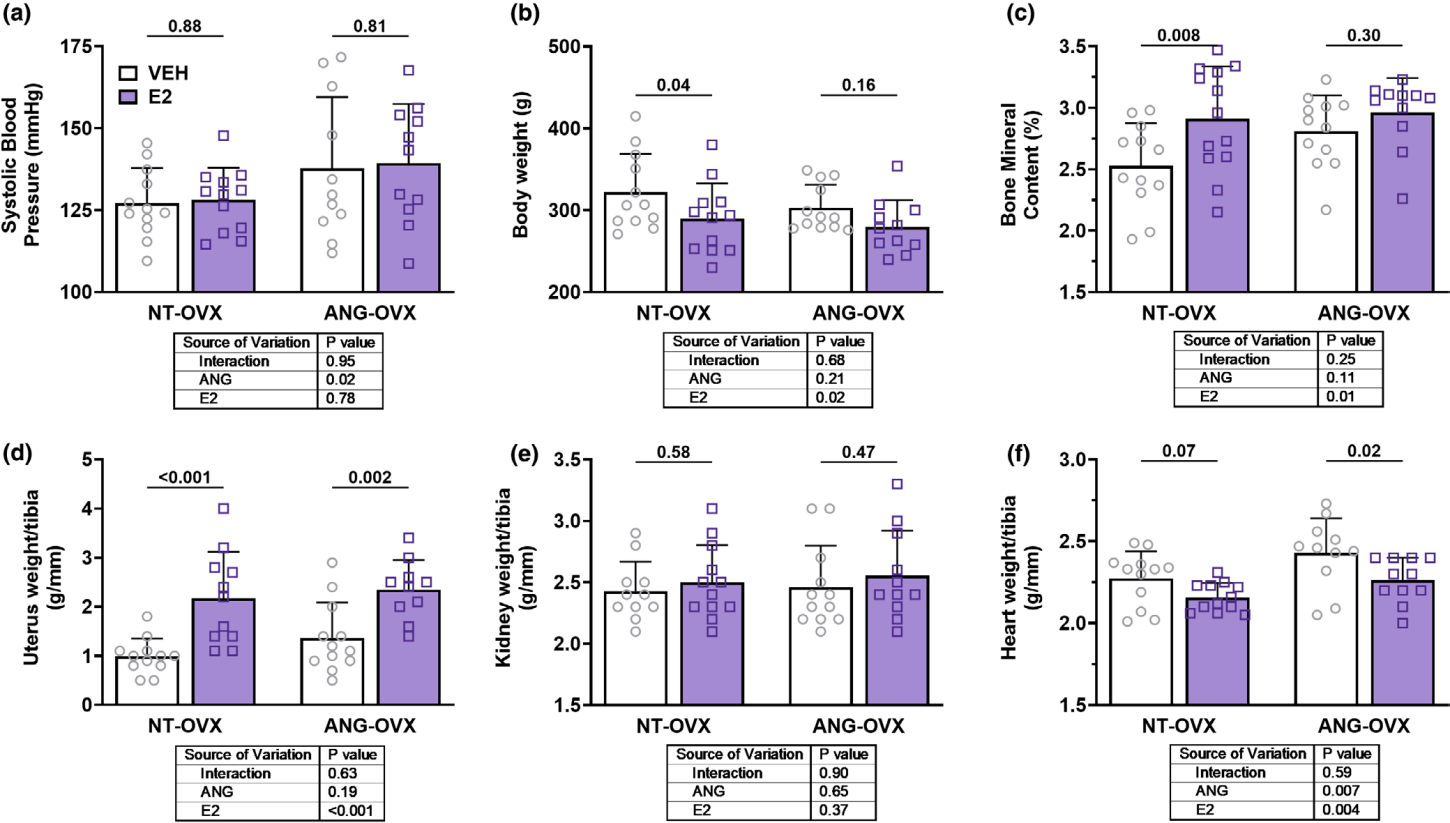
Impact of hypertension and E2 on blood pressure, body composition, and organ weights. (a) Systolic blood pressure (SBP), (b) body weight, (c) bone mineral content percentage, (d) uterus weight/tibia, (e) kidney weight/tibia, and (f) heart weight/tibia in ovariectomized (OVX) rats treated with vehicle (VEH) or estradiol (E2) in either normotensive (NT‐OVX) or hypertensive (ANG‐OVX) conditions, *n* = 10–12 per group. Data presented as mean ± SD. Statistical analysis was performed using two‐way ANOVA.

Systolic blood pressure (SBP) was significantly increased by ANG (*p*_Ang_ = 0.02; Figure 2a) but was not impacted by E2 (*p*_E2_ = 0.78; *p*_int_ = 0.95). Body weight was significantly reduced by E2 (*p*_E2_ = 0.01; Figure 2b), with no significant effect of ANG (*p*_Ang_ = 0.21) or interaction between factors (*p*_int_ = 0.68). Post tests suggest that the main effect of E2 on body weight was largely attributable to the NT‐OVX group (*p* = 0.04), with no significant decrease in the ANG‐OVX group (*p* = 0.16). Bone mineral content was significantly increased by E2 (*p*_E2_ = 0.01; Figure 2c), largely attributable to the effect in NT‐OVX (*p* = 0.008) but not ANG‐OVX (*p* = 0.30). As expected, uterine weight normalized to tibia length was significantly increased by E2 (*p*_E2_ < 0.001; Figure 2d), confirming effective hormone replacement in both groups, with no significant effects of ANG or an interaction. Kidney weight normalized to tibia length did not differ significantly between groups (Figure 2e). In contrast, heart weight/tibia ratio was significantly increased by both ANG and E2 (*p*_Ang_ = 0.007; *p*_E2_ = 0.004), with no significant interaction (*p*_int_ = 0.59). These findings indicate that ANG elevated blood pressure and increased cardiac hypertrophy, E2 reduced body and cardiac weight and enhanced bone mineral content. Complementary physiological data from normotensive and hypertensive ovariectomized rats treated with either vehicle or estradiol are presented in Table 2.

**TABLE 2:** Summary of other measured parameters in middle‐aged ovariectomized female rats.

	NT‐OVX		ANG‐OVX	
	VEH	E2	VEH	E2
Systolic BP (mmHg)*	127 ± 3	128 ± 3	138 ± 7	139.5 ± 5
Diastolic BP (mmHg)*	82 ± 7	84 ± 9	98 ± 25	95 ± 13
Mean arterial pressure (mmHg)*	97 ± 8	98 ± 9.2	114 ± 27	110 ± 14
Pulse pressure (mmHg)	45 ± 6	42 ± 5.8	46 ± 10	44 ± 8.9
Heart rate (bpm)	328 ± 20	329 ± 45	325 ± 35	309 ± 57
Water balance (water intake/urine output)*	3.1 ± 1.2	2.9 ± 1.3	2.5 ± 0.9	2.0 ± 0.7
Urine osmolality (mmmol/kg)*	1576 ± 986	1838 ± 560	1098 ± 369	1038 ± 612
Serum osmolality (mmmol/kg)	317 ± 16	308 ± 12	312 ± 41	303 ± 30
Adrenal weight/tibia (mg/mm)	0.70 ± 0.2	0.86 ± 0.2	0.87 ± 0.2	0.83 ± 0.3

*Note*: Groups include normotensive (NT) and ANG‐infused hypertensive (HT) rats treated with vehicle (VEH) or estradiol (E2), *n* = 10–12 per group. Mean ± SD analyzed by two‐way ANOVA.

* *p*_Ang_ < 0.05.

### 3.3 Urinary parameters

**FIGURE 3:**
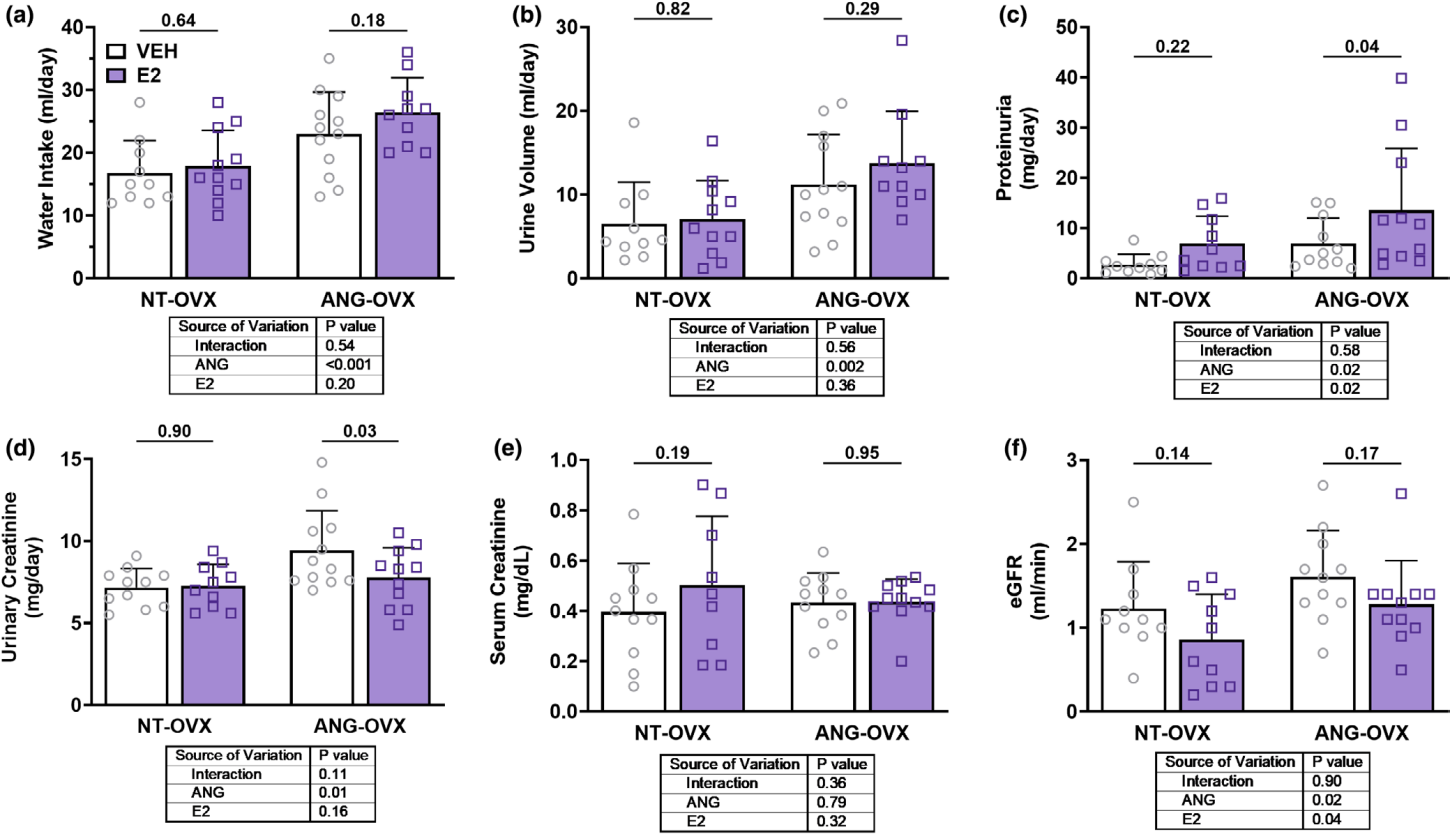
Impact of hypertension and E2 on urinary parameters. (a) water intake; (b) urine volume; (c) proteinuria; (d) urinary creatinine; (e) serum creatinine; (f) eGFR in ovariectomized (OVX) rats treated with vehicle (VEH) or estradiol (E2) in either normotensive (NT‐OVX) or hypertensive (ANG‐OVX) conditions, *n* = 10–12 per group. Data presented as mean ± SD. Statistical analysis by two‐way ANOVA.

Water intake and urine volume were significantly increased in ANG‐treated animals compared with controls (*p*_Ang_ < 0.001 and *p*_Ang_ = 0.002; Figure 3a,b, respectively), while E2 had no significant effect on either parameter (*p*_E2_ = 0.20 and *p*_E2_ = 0.36, respectively). There were no significant interactions between ANG and estrogen treatment for water intake or urine volume (*p*_int_ = 0.54 and 0.56, respectively). As described in the statistical analyses, we examined the simple effects of E2 regardless of the interactions. Proteinuria was significantly increased by both ANG (*p*_Ang_ = 0.02; Figure 3c) and E2 (*p*_E2_ = 0.02), the latter of which was largely attributable to an increase with E2 in the ANG‐OVX group (*p* = 0.04). Urinary creatinine increased with ANG (*p*_Ang_ = 0.01; Figure 3d), and while E2 did not show a main effect, post hoc analysis showed a decrease in this parameter in the ANG‐OVX‐E2 group (*p* = 0.03). Serum creatinine was not different across groups (Figure 3e). The creatinine clearance or eGFR was increased by ANG (*p*_Ang_ = 0.02; Figure 3f) and decreased by E2 (*p*_E2_ = 0.04). Overall, these data suggest that ANG promoted fluid imbalance and renal dysfunction, and E2 enhanced the negative impact of ANG on proteinuria without any effect on the eGFR.

Distinct from the analyses above, we also recorded data before and after OVX for control group animals. Consequently, we performed an additional analysis using paired *t*‐tests of final versus baseline values in the NT‐OVX‐VEH group to assess the effects of OVX alone. OVX significantly increased urine output (3.8 ± 2.4 vs. 6.1 ± 5 mL/day, *p* = 0.02), water intake (13 ± 6.3 vs. 17 ± 5.2 mL/day, *p* = 0.02), and urinary creatinine excretion (5 ± 1.9 vs. 6.7 ± 1.9 mg/day, *p* = 0.05). No significant changes were observed in proteinuria (2.7 ± 2 vs. 2.8 ± 2 mg/day, *p* = 0.19) or blood pressure (132 ± 6 vs. 129 ± 11 mmHg, *p* = 0.36).

### 3.4 Renal histology

**FIGURE 4:**
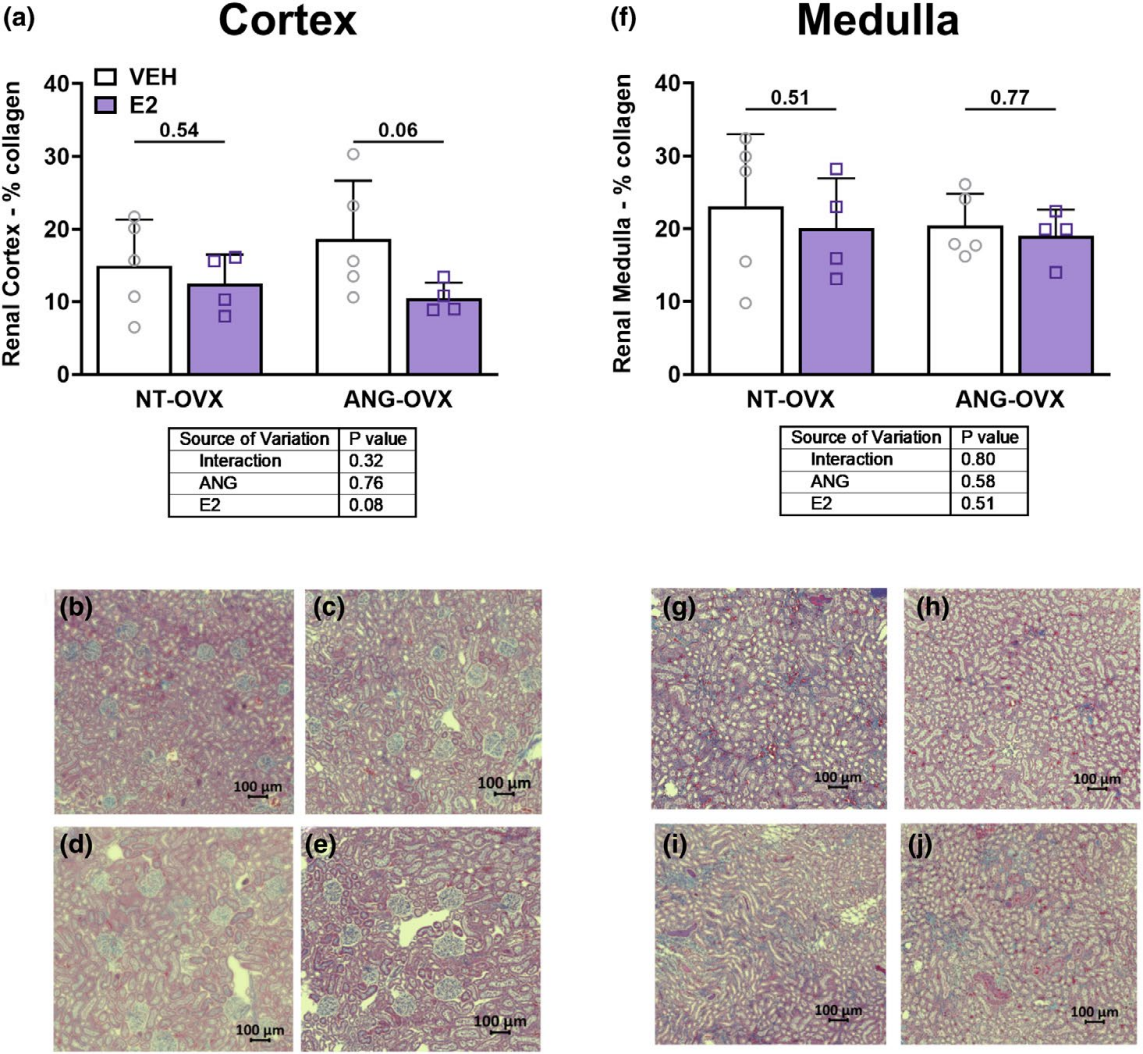
Impact of hypertension and E2 on renal collagen deposition. Collagen deposition in the renal cortex and medulla of ovariectomized (OVX) rats treated with vehicle (VEH) or estradiol (E2) in either normotensive (NT‐OVX) or hypertensive (ANG‐OVX) conditions, *n* = 10–12 per group. Quantification of collagen content in (a) the renal cortex and (f) the renal medulla. Representative trichrome‐stained kidney sections from each group are shown in panels: (b, g) NT‐OVX‐VEH; (c, h) NT‐OVX‐E2; (d, i) ANG‐OVX‐VEH; (e, j) ANG‐OVX‐E2. Data presented as mean ± SD. Statistical analysis was performed using two‐way ANOVA.

Trichrome staining of renal sections found no difference in cortical and tubular collagen deposition between the groups (Figure 4). In fact, there was a trend for reduced collagen with E2, particularly in the ANG‐OVX‐E2 group.

### 3.5 RNA quantification in aorta and kidney

**FIGURE 5:**
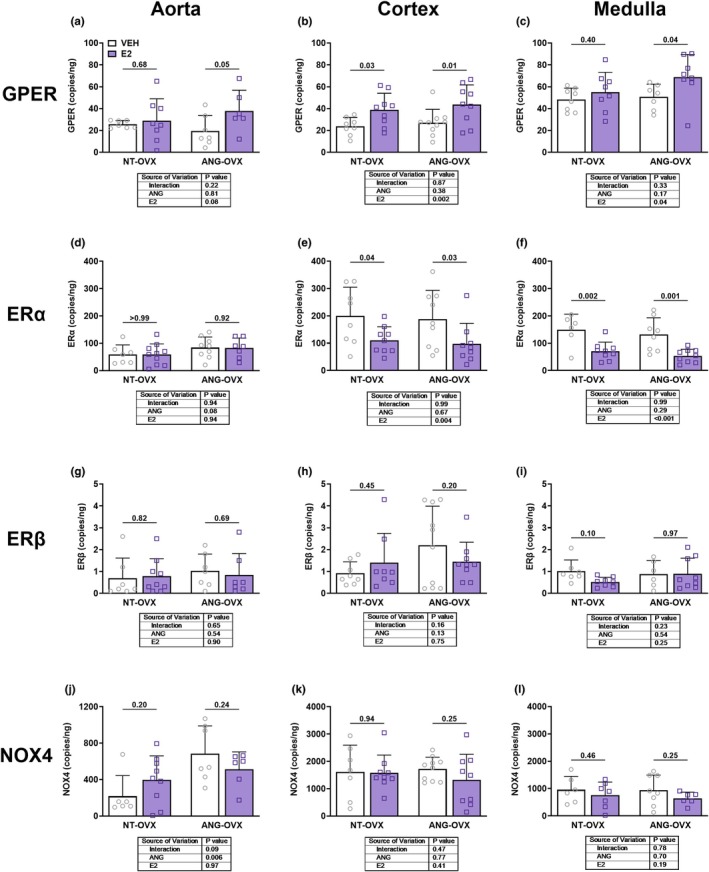
Impact of hypertension and E2 on estrogen receptors and NOX4 expression. Expression of GPER, ERα, ERβ, and NOX4 mRNA in the aorta, renal cortex, and medulla of ovariectomized (OVX) rats treated with vehicle (VEH) or estradiol (E2) in either normotensive (NT‐OVX) or hypertensive (ANG‐OVX) conditions. Columns are labeled with gene names and rows are labeled with tissue. Data presented as mean ± SD. Statistical analysis was performed using two‐way ANOVA.

Next, we quantified mRNA concentration for estrogen receptors GPER, ERα, and ERβ along with NADPH oxidase isoform 4 (NOX4) in aortic and renal lysates (Figure 5). Overall, estrogen receptor expression was not significantly altered by ANG‐induced hypertension in the aorta, renal cortex, or medulla (*p*_Ang_ > 0.1 for all comparisons). However, post hoc analysis revealed that estradiol replacement in the context of hypertension significantly increased GPER mRNA expression in the aorta (*p*_E2_ = 0.08; *p* = 0.05; Figure 5a), cortex (*p*_E2_ = 0.002; *p* = 0.01; Figure 5b), and medulla (*p*_E2_ = 0.04; *p* = 0.04; Figure 5c). In contrast, ERα expression was significantly downregulated by E2 in the cortex (*p*_E2_ = 0.004; Figure 5e) and medulla (*p*_E2_ < 0.001; Figure 5f), regardless of hypertensive status. Specifically, in normotensive animals, E2 reduced ERα expression in both the cortex (*p* = 0.04) and medulla (*p* = 0.03). Similarly, in hypertensive rats, E2 also suppressed ERα expression in the cortex (*p* = 0.002) and medulla (*p* = 0.001). No significant changes were observed in the aorta (*p*_E2_ = 0.94; Figure 5d). ERβ expression was not affected by E2 in any tissue examined: aorta (*p*_E2_ = 0.90; Figure 5g), cortex (*p*_E2_ = 0.75; Figure 5h), or medulla (*p*_E2_ = 0.25; Figure 5i). Regarding oxidative stress, NOX4 expression was significantly upregulated by ANG II in the aorta (*p*_Ang_ = 0.006; Figure 5j), but not in the cortex (*p*_Ang_ = 0.77; Figure 5k) or medulla (*p*_Ang_ = 0.70; Figure 5l). E2 treatment had no effect on NOX4 expression in the aorta (*p*_E2_ = 0.97), cortex (*p*_E2_ = 0.41), or medulla (*p*_E2_ = 0.19), as shown in Figure 5j–l.

## 4 DISCUSSION

This study demonstrates that E2 does not confer protection against ANG‐induced hypertension or renal dysfunction in a middle‐aged menopausal rat model. E2 effectively reduced body weight, increased bone mineral content, and restored uterine weight—confirming the efficacy of hormone replacement—and also conferred cardioprotective effects by reducing heart size. However, E2 did not lower blood pressure, increased proteinuria, and reduced GFR. Despite a trend toward reduced cortical collagen, E2 did not protect against renal damage in response to ANG, suggesting that in the context of preexisting hypertension hormone therapy may aggravate renal injury. Additionally, E2 did not attenuate the ANG‐induced increase in aortic NOX4 expression, suggesting that in this middle‐aged model of menopause E2 is unable to counteract the increase in vascular oxidative stress during hypertension. These findings highlight that the benefits of menopausal hormone therapy are highly context‐dependent and may be detrimental in the presence of established vascular or kidney disease.

In our current model of premenopausal hypertension using middle‐aged, ovariectomized rats, E2 impacted neither blood pressure nor vascular Nox4 mRNA, either in normotensive or hypertensive conditions. In our previous study using normotensive middle‐aged Long Evans rats that were OVX at the same age, blood pressure was elevated 8 weeks after OVX and attenuated by E2 (Zimmerman et al., 2017). Data from Long‐Evans rats who underwent OVX at a similar age of 10 months display increased blood pressure 4 months later (Clark et al., 2004). Since the current study followed OVX for only 4 weeks, the impact of estrogen loss on blood pressure most likely takes longer to develop in this model. More surprising than the lack of E2 effects in the normotensive animals was the inability of E2 to lower blood pressure in the presence of ANG, contrasting multiple studies showing E2‐induced protection from this method of induced hypertension. Despite the lack of an effect on blood pressure, E2 reduced cardiac size, which likely reflects its direct antihypertrophic effects in cardiomyocytes (Calle et al., 2025; Pedram et al., 2005; Wu et al., 2020). Estrogen receptor signaling via GPER and ERβ has been implicated (Di Mattia et al., 2020; Skavdahl et al., 2005; Wang et al., 2017). One of the most striking models of ANG‐dependent and estrogen‐sensitive hypertension is the mRen2.Lewis congenic rat model (Chappell et al., 2003, 2006). In this model, OVX at a young age (4 weeks) significantly increases blood pressure, while OVX at 15 weeks followed by 45 weeks of aging does not impact blood pressure and is renoprotective (Chappell et al., 2008; Yamaleyeva et al., 2007). This latter study shows many similarities to the renal damage in the presence of estrogen in the current study, including increased proteinuria and reduced GFR, which is associated with increases in circulating renin, ACE, and ANG. Although not assessed in this study, OVX upregulates while estrogen replacement suppresses AT1a receptor expression in the kidney and aorta (Harrison‐Bernard et al., 2003; Macova et al., 2008; Nickenig et al., 1998; Owonikoko et al., 2004; Rogers et al., 2007). We also probed for Nox4 in the vasculature since our previous work shows that selective GPER activation reduces hypertension‐associated oxidative stress in the aorta and kidney and that genetic deletion of GPER removes female protection against ANG‐induced oxidative stress and vascular stiffening primarily through downregulation of Nox4 (Lindsey et al., 2011; Liu et al., 2016; Ogola et al., 2019). In the current study, administration of E2 to simulate clinical treatments for menopause did not lower vascular Nox4, which may be attributed to the different treatment regimen or the age difference between these studies. Taken together, the inability of E2 to modulate blood pressure in the current study is most likely related to the timing of OVX at middle age to mimic human menopause, the short time course (4 weeks) after OVX, or the use of E2 rather than a receptor‐selective approach.

ANG‐induced hypertension increased GFR while E2 lowered this value, indicating that ANG and/or a mild increase in blood pressure enhanced GFR in this model. While hypertensive models such as the spontaneously hypertensive rat display a decrease in GFR, fawn hooded hypertensive rats have greater GFR compared with normotensive controls (de Keijzer et al., 1988), which may result from a mutation in γ‐adducin that impairs autoregulation (Fan, Gao, et al., 2020b; Fan, Geurts, et al., 2020a). Increased GFR in response to 6‐week ANG infusion is observed in male Wistar rats and is attributed to impaired autoregulation, decreased renal vascular resistance, and increased renal blood flow (Casare et al., 2016). Acute ANG influences the constriction of both afferent and efferent arterioles, and the sensitivity of each ultimately determines renal perfusion (Gupta, 2018). With prolonged exposure, however, ANG contributes to glomerular damage and eventual GFR decline (Maranduca et al., 2023). The increase in GFR could also result from the dipsogenic impact of ANG in the brain (Harland et al., 1988), noted by increased water intake in the current study. Urine output exceeded the increased water intake while urine osmolality significantly decreased, a pattern also noted in ANG‐infused OVX mice (Dutta et al., 2023).

In our previous study in normotensive rats, E2 treatment for 80 days after midlife OVX maintains lower blood pressure and plasma glucose but induces renal hypertrophy, elevates proteinuria, and reduces GFR (Zimmerman et al., 2017). We replicated that data in the current study, finding that E2 for 28 days after midlife OVX similarly elevated proteinuria and reduced GFR. Baseline GFR is not changed 5 months after OVX at 8 months of age (Nielsen et al., 2003), while estrogen‐intact conditions are associated with better renal function 9 months after OVX at 10 or 24 weeks of age (Pijacka et al., 2015; Yousefzadeh et al., 2023). These differing outcomes based on the timing of OVX may indicate that ovarian hormones during development are necessary to protect the kidney but acquire a neutral or detrimental role during aging. Hyperfiltration is also noted in diabetic female mice and mediated by estrogen receptor ERα66 (Irsik et al., 2018). In healthy postmenopausal women, hormone therapy significantly increases GFR while other renal markers remain stable, suggesting a protective effect on kidney function (Kaygusuz et al., 2012). In contrast, a large cohort study in women over 66 years of age found that hormone therapy, particularly oral estrogen, was independently associated with a dose‐dependent decline in kidney function, highlighting potential risks of estrogen use in older women (Ahmed et al., 2008). These findings underscore the complex interplay between hormonal status and ANG‐induced hypertension in regulating renal function.

Proteinuria is not only a marker of kidney damage but also a direct contributor to disease progression by activating proinflammatory and profibrotic pathways that drive chronic tubulointerstitial injury (Gorriz & Martinez‐Castelao, 2012; Stevens et al., 2024). In our model, although no significant differences in cortical or medullary collagen deposition were observed, the combination of hypertension and E2 markedly increased proteinuria. The rise in proteinuria may reflect E2's catabolic or hyperfiltration effects rather than direct renal injury, since E2 reduces body weight and enhances energy expenditure (Mauvais‐Jarvis et al., 2013) and increases filtration without structural damage (Guldan et al., 2024). Similar findings are reported in female Wistar rats treated with L‐NAME and ANG, where both ovary‐intact and OVX + E2 groups develop renal injury and proteinuria while OVX rats without estrogen replacement are protected (Oestreicher et al., 2006). Similarly, in Dahl salt‐sensitive rats with heart failure E2 exacerbates renal damage after OVX by promoting microvascular and glomerular damage, even though it suppresses components of the renin‐angiotensin system (Hoshi‐Fukushima et al., 2008). Our previous work also showed that long‐term E2 treatment increased proteinuria in OVX Long‐Evans rats, and that co‐administration of medroxyprogesterone protected against the E2‐induced renal dysfunction (Zimmerman et al., 2017, 2020). However, opposite results are found in other animal models such as salt‐loaded mRen2.Lewis rats, where OVX worsens and E2 protects both hypertension and proteinuria (Chappell et al., 2006; Cohen et al., 2010). These conflicting results may be attributed to differences in disease models, age at intervention, timing of OVX, and variations in hormone type, dose, and delivery. The inconsistency across studies highlights the incomplete understanding of E2's actions on the kidney and underscores the need for further research to clarify its role in renal outcomes under pathological conditions.

Our group previously established absolute estrogen receptor levels using ddPCR in tissues from both rat and mouse (Gurrala et al., 2021; Hutson et al., 2019). Similar to previous findings, we found in the current study that ERα was the highest expresser, GPER second, while ERβ was expressed at very low levels. Tissue‐specific expression was similar for GPER and ERβ while ERα expression was significantly higher in kidney versus aorta. Interestingly, the only negative feedback noted was renal ERα, significantly decreasing with E2 treatment in both the cortex and medulla but not the aorta. In contrast, GPER was upregulated by E2 in all tissues, especially during ANG‐induced hypertension. Negative feedback regulation is commonly found in endocrine systems and demonstrated by a decrease in ERα in response to E2 treatment in MCF‐7 breast cancer cells (Saceda et al., 1988) as well as in human internal mammary arteries, where ERα is downregulated along with ERβ and GPER (Haas et al., 2007). In contrast, E2‐induced stimulation of ERα, ERβ, and GPER is found in other types of cancer (Pena‐Gutierrez et al., 2022; Vladusic et al., 2000). Tissue‐specific regulation is best demonstrated in the brain, where E2 decreases hypothalamic ERα mRNA in ventromedial and arcuate but not dorsomedial nuclei and is dependent on the dosage of E2 (Lauber et al., 1990, 1991). Taken together, transcriptional regulation of estrogen receptors in response to the primary ligand E2 varies widely depending on tissue type and should not be assumed to occur in only one direction.

This study utilizes a clinically relevant midlife menopausal model to test the impact of E2 on preexisting hypertension. Since the current treatment regimen restores physiological E2 concentrations to levels seen during proestrus (Bohacek & Daniel, 2007, 2010), we do not think that the negative renal effects in the current study are due to supraphysiological levels of E2. Clark et al. (2004) also used silastic capsules for administration and found E2 levels of ~75 pg/mL, and Chappell et al. (2003) used subcutaneous pellets to reach serum levels of 194 ± 79 pg/mL, yet neither of these papers reports negative impacts on the kidney. However, the short treatment duration may limit our understanding of renal damage, and the animal model, while controlled, may not fully reflect human physiology. Future studies should extend treatment periods and examine specific estrogen receptor pathways that protect vascular health without harming the kidneys. Although ovariectomy is a common model for menopause research in rodent models, it induces abrupt hormone loss which does not mimic the gradual transition of human menopause. The VCD model, which induces follicle depletion while preserving the ovaries, offers a more physiologically relevant alternative (Brooks et al., 2016). Although telemetry is the most accurate method for measuring blood pressure, our data align well with our prior findings and those from similar studies (Clark et al., 2004; Zimmerman et al., 2017, 2020). Future studies should incorporate telemetry into rat models to investigate how OVX and E2 impact 24 h blood pressure, including circadian blood pressure rhythms.

Taken together, our findings underscore that menopause, cardiovascular disease, and aging may diminish or impair the cardiovascular benefits typically associated with E2. These data may reflect findings from the Women's Health Initiative, where extrapolation by age reveals that conjugated equine estrogens were protective against coronary heart disease in women aged 50–59, neutral in those aged 60–69, and detrimental in women over 70 (Manson et al., 2013). Moreover, the impact of E2 in midlife OVX rats was organ‐specific and therefore findings should not be extrapolated from one tissue to another, especially in the presence of cardiovascular or other diseases. While E2 may provide cardiovascular benefits under certain conditions, its impact on renal health is highly dependent on the presence of comorbidities such as hypertension. These findings provide support for both the “timing hypothesis” and “healthy cell bias” hypotheses, which suggest that timing and underlying disease status critically influence the efficacy of menopausal hormone therapy (Brinton, 2005; Clarkson et al., 2013). In addition, these results highlight the need for a more personalized approach to menopausal hormone therapy and support further investigation into targeted strategies that preserve vascular function without exacerbating renal injury.

## AUTHOR CONTRIBUTIONS

APOL performed experiments, interpreted results of experiments, prepared figures, drafted the manuscript, edited and revised the manuscript. IPDS, YZ, SAB, HP, NJ, HCC, RM, and ABW performed experiments and approved the final version of the manuscript. JMD conceived and designed the research, interpreted the results of the experiments, approved the final version of the manuscript. SHL conceived and designed research, analyzed data, interpreted results of experiments, prepared figures, edited and revised the manuscript.

## FUNDING INFORMATION

This work was funded by National Institutes of Health P01 AG071746 (JMD, SHL).

## CONFLICT OF INTEREST STATEMENT

No financial conflicts to disclose.

## ETHICS STATEMENT

This study complied with the National Research Council (US) Committee for the Update of the Guide for the Care and Use of Laboratory Animals (2011), and all procedures received approval from the Tulane Institutional Animal Care and Use Committee.
